# Facet joint degeneration in adolescent idiopathic scoliosis

**DOI:** 10.1002/jsp2.1016

**Published:** 2018-05-24

**Authors:** Daniel G. Bisson, Polly Lama, Fahad Abduljabbar, Derek H. Rosenzweig, Neil Saran, Jean A. Ouellet, Lisbet Haglund

**Affiliations:** ^1^ Orthopaedic Research Laboratory Shriners Hospital for Children Montreal Quebec Canada; ^2^ Department of Orthopedic Surgery McGill University Montreal Quebec Canada; ^3^ Department of Orthopedic Surgery King Abdulaziz University Jeddah Saudi Arabia

**Keywords:** cartilage, cell proliferation, cytokines, extracellular matrix, facet joint, metalloproteases, osteoarthritis, proteoglycans, scoliosis

## Abstract

Adolescent idiopathic scoliosis (AIS) is a poorly understood deformity of the thoracolumbar spine which affects the intervertebral discs (IVDs) and the articular facet joints. The knowledge concerning facet joints in this context is very limited, although facet joint degeneration is a known contributor of back pain. In this study, a comprehensive investigation was performed to characterize the facet joint chondrocytes and extracellular matrix within the scoliotic spine. Surgically removed articular facet joint tissues were collected from patients undergoing spinal corrective surgery for AIS deformities, while non‐scoliotic articular facet joint tissues were obtained from cadaveric organ donors. Alterations in cartilage tissue structure were evaluated histologically with safranin‐O fast green and a modified OARSI grading scale. Pro‐inflammatory cytokines, matrix‐degrading proteases, and fragmented matrix molecules associated with cartilage degradation were analyzed by immunohistochemistry and western blotting. Safranin‐O fast green staining revealed that young scoliotic facet joints show clear signs of degeneration with substantial proteoglycan loss, similar to osteoarthritis (OA). The proteoglycan levels were significantly lower than in healthy asymptomatic non‐scoliotic control individuals. In comparison to controls, scoliotic articular facets showed increased cell density, increased expression of the proliferation marker Ki‐67, and higher expression of MMP‐3, MMP‐13, and IL‐1β. Expression and fragmentation of the small leucine‐rich proteins (SLRPs) chondroadherin, decorin, biglycan, lumican, and fibromodulin were analyzed with western blot. Chondroadherin and decorin were fragmented in cartilage from patients with a curve greater than 70°, whereas biglycan and fibromodulin did not show curve‐related fragmentation. AIS facet joint cartilage shows hallmarks of OA including proteoglycan loss, overexpression of pro‐inflammatory mediators, increased synthesis of matrix‐degrading proteases and fragmentation of SLRPs. As with patients with age‐related OA, the premature joint degeneration seen in scoliotic patients is likely to contribute to the pain perceived in some individuals.

**ABBREVIATIONS**
AIS: adolescent idiopathic scoliosis
IVD: intervertebral discs
MMP: matrix metalloproteases
OA: osteoarthritis
SLRP: small leucine‐rich proteins

## 1 INTRODUCTION

Adolescent idiopathic scoliosis (AIS) is a structural deformity which manifests as a gradually increasing curvature and rotation of the spine.^1^ Patients with AIS can experience mild discomfort which later progresses as impairment in spinal function and impaired breathing due to reduced area for lungs to expand.^2^ There is a worldwide prevalence of 3% of children aged 10‐16 years who suffer from AIS,^3^ and currently there are two treatment options: (1) bracing if the curve is below a 50° Cobb angle (major curve angle) and (2) spinal fusion with instrumentation when the curvature is greater than a 50° Cobb angle.^2^ Although the treatment options are highly beneficial for managing progression, the long‐term effects on patient's quality of life are still unclear.^4^

The clinical system used to classify the wide range of curve types in AIS is called the Lenke system, developed by Lawrence Lenke and colleagues.^5^ It provides reliable two‐dimensional classification using upright coronal and sagittal radiographs to determine (1) Curve type (1‐6) (2) Lumbar spine modifier (A, B, C), and (3) Sagittal thoracic modifier (−, N, +). This system allows for an objective classification of every possible curve pattern in scoliosis and helps clinicians choose the best treatment accordingly.

In non‐scoliotic individuals, the symmetry of the spine allows its load‐bearing to be balanced between the intervertebral discs (IVD) and the corresponding facet (zygapophyseal) joints.^6^ Due to the midline location of IVDs and their large size, 70%‐80% of the load is transmitted through the IVDs and vertebral bodies.^7^ The facet joints compensate for the remaining 20%‐30% of load distribution. The bilateral position of facet joints gives them the role of limiting flexion and torsion of the spine.^8^ In patients with AIS, this equilibrium is shifted and consequently the facet joints become subjected to increased and unbalanced loads because of the abnormal torsion and rotation of the spinal column. It is well‐known that load magnitude affects bone and cartilage development and growth as well as proteoglycan production by chondrocytes.^9^ Thus, inadequate or excessive loading has detrimental effects on cartilage matrix metabolism.^10^ Apart from traumatic injury, which is the most obvious form of abnormal biomechanics, slight shifts in ambulatory mechanics,^11^ abnormal tension, shear forces, and malalignment of articular joints^12^ are known to contribute toward cartilage degeneration. In fact, load‐induced changes are one of the triads of primary osteoarthritis, along with genetic influences and age‐related changes.^10^ In consequence of abnormal spinal curvatures and loading, osteoarthritis (OA) (defined as destruction of articular cartilage and subchondral bone) could be present in the facet joints of younger AIS patients. Osteoarthritis is also known to be prevalent in 15%‐45% of the facet joints in patients with chronic low back pain.^13^

Cell proliferation and formation of cell clusters can often be observed in OA. The chondrocytes overexpress pro‐inflammatory cytokines, such as IL‐6, IL‐1β, and matrix‐degrading enzymes, such as MMP‐3 and MMP‐13.^14^ Increases in these factors shift matrix homeostasis toward catabolism, thereby promoting tissue destruction. Consequentially, the cartilage loses proteoglycan content early along with its collagen network later in the disease process.^15^ The small leucine‐rich proteins (SLRPs), which include biglycan, decorin, chondroadherin, fibromodulin, and lumican,^15^ have also been reported to be proteolytically fragmented in OA.^16^ SLRPs are found in the pericellular or territorial matrix ,^17^ and their fragments may potentially act as alarmins^18^ by activating toll‐like receptors TLR 2 and 4 in articular cartilage. TLR activation triggers the secretion of pro‐inflammatory mediators.^19^

The aim of this study is to investigate the effects of scoliotic curvature on the articular facet joint homeostasis in patients with AIS. Our underlying hypothesis is that abnormal curvature of the scoliotic spine results in degenerative changes and increased secretion of cytokines and proteases in facet joints, as reported in osteoarthritis.

## 2 MATERIALS AND METHODS

### 2.1 Sample collection

Multiple superior articular facet joints were collected from AIS patients undergoing spinal fusion corrective surgery after consent. The facet joints collected span multiple spinal levels with the levels collected depending on the surgical requirement. The patient's spinal curves were classified by the orthopedic surgeons according to their major curve types using the Lenke system, which categorizes all patients in 6 major curve types with subclassifications for lumbar and sagittal thoracic modifiers using bi‐planar radiographs (Table 1).^5^ Articular facet cartilage samples of 20 scoliotic patients, age range 11‐19 years, with a mean age of 15.23 ± 2.36 were collected. Of which, 75% were from female patients (Table 2). The study was approved by the institutional review board of McGill University (IRB # A00‐M113‐13B) Montreal, Canada.

**Table 1:** Lenke classification system for scoliositic spine curvatures

Lenke type	Curve type	Description
1	**Main thoracic (MT)**	**Thoracic side‐bending cobb > 25°**
2	**Double thoracic (DT)**	**MT + T2‐T5 kyphosis > 120°**
3	**Double major (DM)**	**MT + T10‐L2 kyphosis > +20°**
4	**Triple major (TM)**	**MT + DT + DM**
5	**Thoracolumbar/lumbar (TL/L)**	**Lumbar side‐bending cobb > 25°+ T10‐L2 kyphosis > 20°**
6	**Thoracolumbar/lumbar – Structural MT**	**MT (minor) + TL/L**

**Table 2:** Scoliotic and non‐scoliotic donors used with age, sex, Lenke type (scoliotic only), major curve cobb angle (scoliotic only), and cause of death (non‐scoliotic only)

Group	Age	Sex	Lenke type	Major curve cobb angle (°)	Cause of death
Scoliotic	18	**F**	**1**	**56**	
Scoliotic	**19**	**M**	**6**	**53**	
Scoliotic	**18**	**M**	**5**	**55**	
Scoliotic	**15**	**F**	**1**	**60**	
Scoliotic	**16**	**M**	**4**	**80**	
Scoliotic	**18**	**F**	**5**	**50**	
Scoliotic	**12**	**F**	**4**	**100**	
Scoliotic	**16**	**M**	**1**	**58**	
Scoliotic	**12**	**F**	**3**	**60**	
Scoliotic	**16**	**F**	**1**	**48**	
Scoliotic	**17**	**F**	**5**	**60**	
Scoliotic	**17**	**M**	**2**	**85**	
Scoliotic	**12**	**F**	**4**	**75**	
Scoliotic	**14**	**F**	**5**	**50**	
Scoliotic	**13**	**F**	**2**	**70**	
Scoliotic	**12**	**F**	**1**	**48**	
Scoliotic	**13**	**F**	**6**	**67**	
Scoliotic	**17**	**F**	**1**	**45**	
Scoliotic	**14**	**F**	**1**	**50**	
Scoliotic	**17**	**F**	**1**	**55**	
Non‐Scoliotic	**50**	**M**			**Anoxia**
Non‐Scoliotic	**50**	**M**			**Stroke**
Non‐Scoliotic	**28**	**M**			**Anoxia**
Non‐Scoliotic	**17**	**M**			**Anoxia**
Non‐Scoliotic	**34**	**F**			**Stroke**
Non‐Scoliotic	**27**	**M**			**Trauma**

Through a collaboration with Transplant Quebec, 6 asymptomatic non‐scoliotic articular facet joint pairs from the thoracolumbar region, age range 17‐60 years, with a mean age of 34.33 ± 13.31 were obtained from cadaveric organ donors who had no history of back pain or other spinal deformities (Table 2).

### 2.2 Tissue processing for explant culture

Articular cartilage was separated from the underlying subchondral bone using a scalpel. Cartilage explants were washed with phosphate‐buffered saline (PBS) containing 50 μg/mL gentamycin (Life Technologies), 0.5 μg/mL amphotericin B (Life Technologies) for 5 minutes. Cartilage explants were kept in Dulbecco's modified eagle medium (DMEM) with 4.5 g/L glucose (Sigma), 25 μg/mL gentamycin (Life technologies), 2 mM Glutamax (Life Technologies), and 10% fetal bovine serum for 24 hours following dissection. The facets were cultured for 96 hours in serum‐free DMEM, 25 μg/mL Gentamycin (Life Technologies), 1× Glutamax (Life Technologies), Insulin‐transferrin‐selenium (ITS, Life Technologies), 50 μg/mL ascorbic acid on volume per weight basis (10×, v/w) at 37°C, 5% CO_2_.

### 2.3 Histology

Dissected facet cartilage was fixed in 4% paraformaldehyde overnight, followed by sequential immersion in 10%, 20%, and 30% sucrose for 12 hours each before OCT embedding. Cryosectioning was performed on a CryoStar NX70 cryostat (ThermoFisher Scientific), and 12‐μm‐thick sections retrieved were arranged on charged Superfrost Plus Slides (VWR). Prior to staining, slides were heated at 60°C for 15 minutes and rehydrated in PBS‐T (0.05% Triton‐x) for 5 minutes. Safranin‐O (0.025%) (Sigma Aldrich, Canada) and fast green stain (0.01%)(Sigma Aldrich, Canada) were applied to the sections for 5 minutes each as mentioned in published protocols,^20^ and a semi‐quantitative grading was performed on the tissue sections.

### 2.4 Immunohistochemistry

The 12‐μm‐thick sections were immunostained for proliferative and pro‐degenerative factors. The primary antibody to the proliferative marker Ki‐67 (10 ng/mL) (NB110‐89717) (NovusBio) was applied at 1:100 dilution, while antibodies to pro‐inflammatory markers IL1ß (ab9722) (2.5 μg/mL), IL6 (5 μg/mL) (ab9324) and matrix‐degrading enzymes MMP3 (2 μg/mL) (ab52915), MMP13 (5 μg/mL) (ab39012), (Abcam) were used at a 1:200 dilution for 1 hour at room temperature. Followed by PBS washes, diaminobenzidine (DAB) chromogen (Abcam) staining kit was used to identify the immunopositive cells. These cells were counted from images captured under ×20 and ×40 objectives with Olympus DP70 digital camera (Olympus) pre‐fixed to a Leica microscope (Leica DMRB) under visible light.

### 2.5 Histology staining and quantification

A modified OARSI grading scheme (Grade 0‐4) was used and grading of the safranin‐O fast green‐stained sections was performed blinded by 3 independent evaluators.^21^ Healthy cartilage is given a grade 0, and OA starts at grade 1, where mild abrasion and proliferation can occur. At grade 2, there is discontinuity in the cartilage surface that can transform into fissures at grade 3. At grades 4 and 4.5, erosion and excavation takes place, respectively. OARSI grades 5 and 6 (characterized by denudation, deformation, and bone remodeling) were excluded since the cartilage was removed from the bone before analysis. For cell counts, DAPI (Vectashield) and Mayer's hematoxylin (Sigma) stained cell nuclei were counted and normalized to cartilage area using ImageJ software (NIH). All slides were examined using the ×5 objective. A MATLAB script was developed with background normalization, and the area selection tool was used to assess the proteoglycan content through average red pixel intensity in a semi‐quantitative manner.^22^ In the MATLAB script, the red staining intensity of the entire section was quantified by normalizing to the white background and extracting the RGB (red‐green‐blue) intensities of each pixel in the region of interest, which was drawn over the cartilage and then isolating the red channel only which was finally averaged over the entire region of interest.

### 2.6 Protein extraction and digestion

Articular cartilage was finely minced, and proteins were extracted at 4°C under continuous agitation for 72 hours using 15 volumes per gram of tissue of 4 M guanidine hydrochloride (Sigma Aldrich), 50 mM sodium acetate and 10 mM EDTA (Sigma–Aldrich, Canada), and COMPLETE protease inhibitors (Roche), pH 5.8. The extract was separated from the tissue by centrifugation for 30 minutes at 13 000*g*, and aliquots of 8 μL of were prepared for SDS‐PAGE. Samples were treated with keratanase (Amsbio LLC) and chondroitinase ABC (Ambsio LLC) prior to SDS‐PAGE and western blotting.^16^

### 2.7 Western blotting

Proteins were fractionated on 10% SDS‐PAGE gels and were transferred to a nitrocellulose membrane. The membrane was blocked with 3% skim milk powder in 0.01 M Tris‐HCl, 0.15 M NaCl, 0.1% Tween20 (TBS‐T), pH 7.6. Proteins were detected by chemiluminescence (GE Healthcare) and in‐house generated antibodies to biglycan, lumican, chondroadherin, decorin, fibromodulin.

### 2.8 Statistics

Unpaired and paired parametric student *t* test were performed in GraphPad (Prism) to assess significance valued at *P* < .05. Confidence intervals of 95% were used to plot error bars.

## 3 RESULTS

### 3.1 Histology and OARSI score

**Figure 1:**
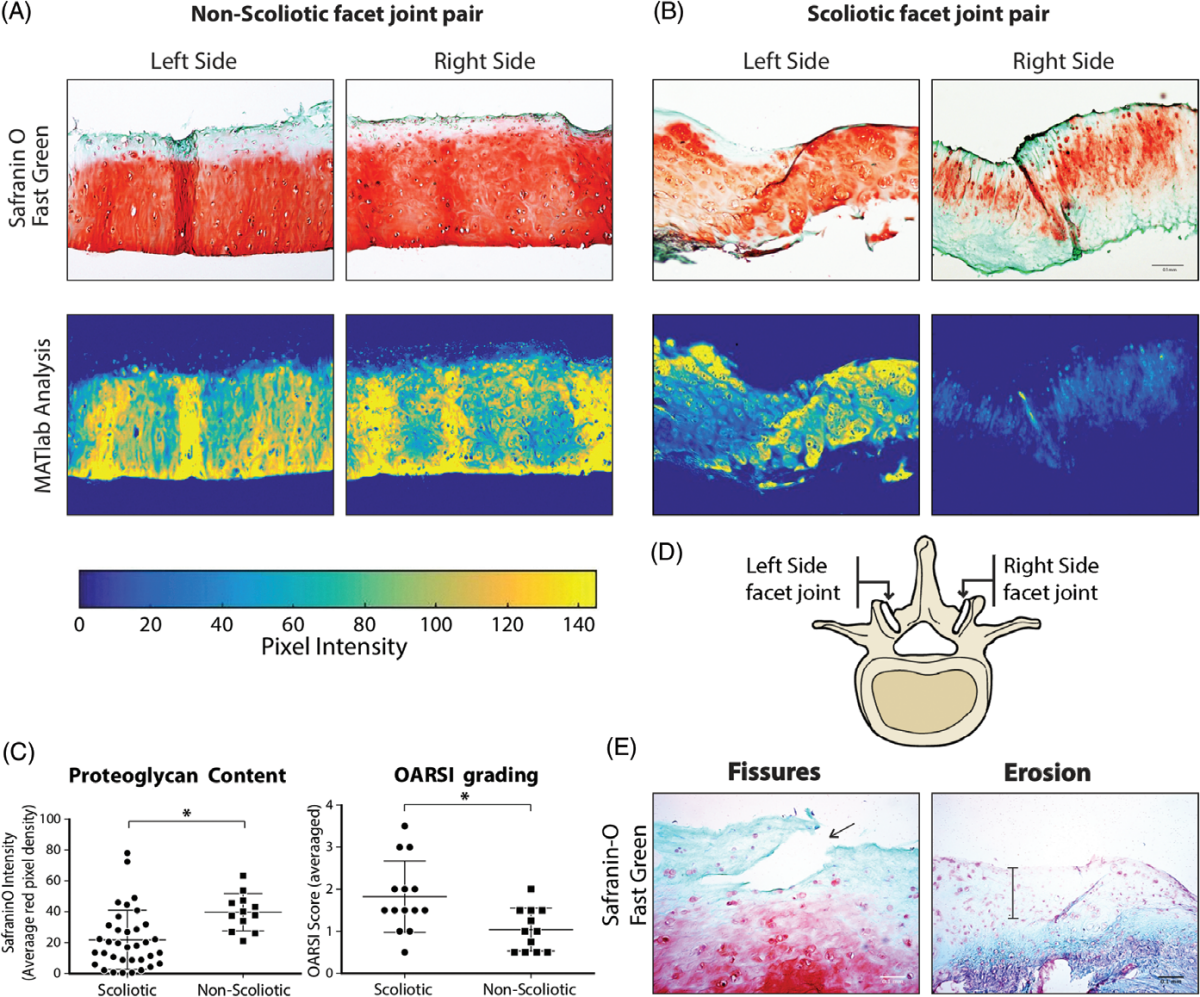
Safranin‐O fast green staining of bilateral facet joint cartilage from A, Scoliotic and B, Non‐scoliotic donors with red staining quantification using MATlab. C, Overall safranin‐O intensity and OARSI grading comparison between Scoliotic and non‐Scoliotic donors. D, Schematic representing a facet joint pair from the same vertebrae. E, Osteoarthritic markers found in scoliotic cartilage and highlighted by arrow (fissure) and thinning (erosion). Error bars shown are 95% CI. Unpaired parametric student's *t* test (**** = *P* < .0001, * = *P* < .05)

**Figure 2:**
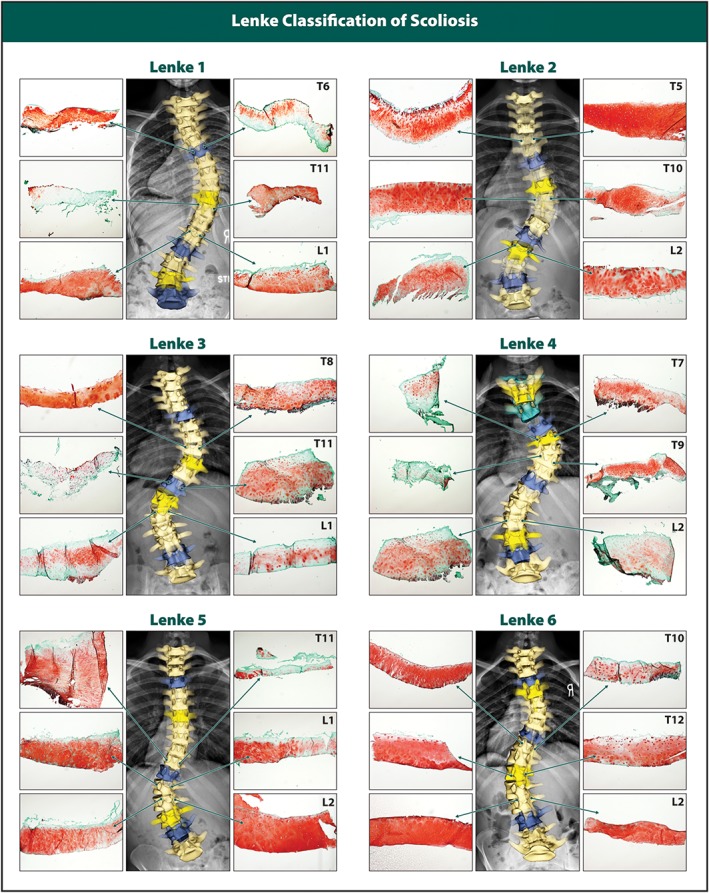
Safranin‐O fast green staining of articular cartilage from 3 facet joint pairs (left and right side for 3 spinal levels for 6 samples overall) for each of 6 Lenke types

Articular facet joint cartilage from both scoliotic and non‐scoliotic groups was stained with safranin‐O fast green to assess proteoglycan levels and overall cartilage tissue condition. Hallmarks of osteoarthritis in the form of cartilage fibrillation and erosion (Figure 1D) were observed in a few samples, while proteoglycan loss was evident mainly from the superficial zone of the scoliotic tissue sections. To assess proteoglycan content semi‐quantitatively, a MATLAB script (Figure 1A,B) was used to calculate the mean red pixel intensity of the safranin‐O stain of the entire section. The scoliotic facet joint cartilage had significantly (*P* < .0001) lower pixel intensity compared to the non‐scoliotic group. The scoliotic facet cartilage had an average OARSI grade of 2.14 ± 0.81, and the non‐scoliotic had an average OARSI grade of 1.04 ± 0.51. The difference was significant (*P* < .05), with the scoliotic cartilage showing a full grade point higher average score (Figure 1C). Safranin‐O fast green staining intensity revealed variable proteoglycan content between the two sides in a facet joint pair in scoliotic spines (Figure 2), whereas no difference was detected in the non‐scoliotic group (Figure 1A)**.**

### 3.2 Cell density and proliferation

**Figure 3:**
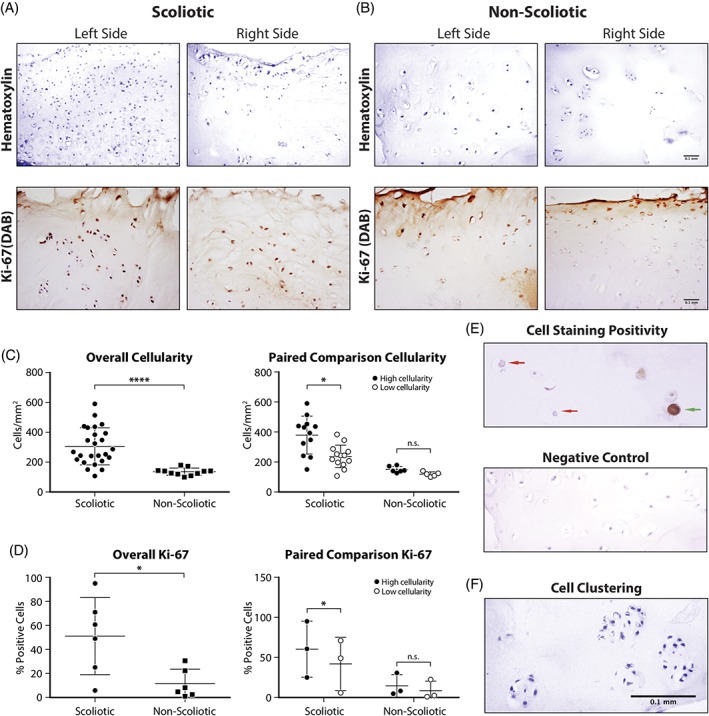
Cell density and proliferative marker Ki‐67 IHC within facet joint cartilage from A, AIS and B, Non‐Scoliotic groups revealed by hematoxylin and DAB staining respectively. C, Cell density quantification between scoliotic and non‐scoliotic groups as well as paired comparison in high cellularity and low cellularity subgroups within the same donors. D, Ki‐67 immunopositivity quantification and comparison between scoliotic groups and the same cellularity subgroups as C). E, High magnification Ki‐67 IHC to show positive cells (green arrow) and negative cells (red arrow) and negative control (secondary antibody only) F, Image of scoliotic facet joint cartilage showing cell cluster, as revealed by hematoxylin. Error bars shown are 95% CI. Unpaired *t* test for overall comparisons and paired *t* test for paired comparisons (**** = *P* < .0001, * = *P* < .05)

The number of chondrocytes in the cartilage was determined by labeling cell nuclei with hematoxylin. Scoliotic cartilage had a significantly (*P* < .0001) higher number of cells, averaging 1.5‐fold more compared to non‐scoliotic cartilage (Figure 3C)**.** Some scoliotic cartilage samples also showed more cell clustering, although this was not seen in all samples. Cell numbers were very similar between the two facet joints from the same spine level in every non‐scoliotic donor. Side‐to‐side differences in cell number were often observed in the scoliotic cartilage at the same spine level. To determine if the cause for higher cell count was due to proliferation, as reported in osteoarthritis,^23^ the proliferative marker Ki‐67 was used for immunohistochemical analysis. Ki‐67 positive cell numbers were significantly (*P* < .0001) greater in all scoliotic samples compared to non‐scoliotic and always correlating with higher cell density when compared side‐to‐side (Figure 3C)***,*** with a high number of proliferating cells in the clusters (Figure 3F)**.**

### 3.3 Degenerative factor expression

**Figure 4:**
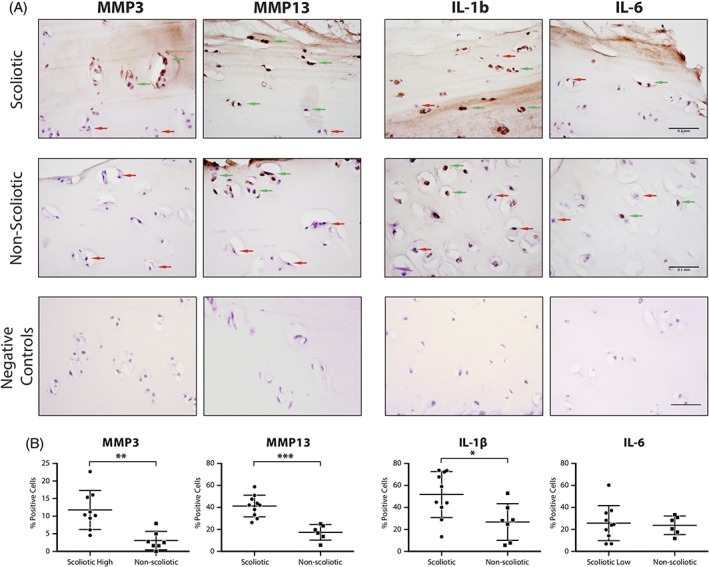
Scoliotic and non‐scoliotic cartilage immunohistochemistry of proteases MMP‐3, MMP‐13, and pro‐inflammatory cytokines IL‐1β and IL‐6 with cell‐positivity quantification. Positive cells are shown by green arrows and negative cells by red arrows. Error bars shown are 95% CI. Unpaired *t* test (*** = *P* < .001, ** = *P* < .01, * = *P* < .05)

Immunohistochemical staining showed a significant (*P* < .001) increase in positive staining for MMP‐3, −13, and IL‐1ß in the scoliotic tissue **(**Figure 4). Positive cells were defined by strong intracellular and pericellular brown stain, which was normalized to all cells counterstained with hematoxylin. IL‐6 showed the weakest immune reactivity with no difference between control and scoliotic groups.

### 3.4 Fragmentation of SLRPs

**Figure 5:**
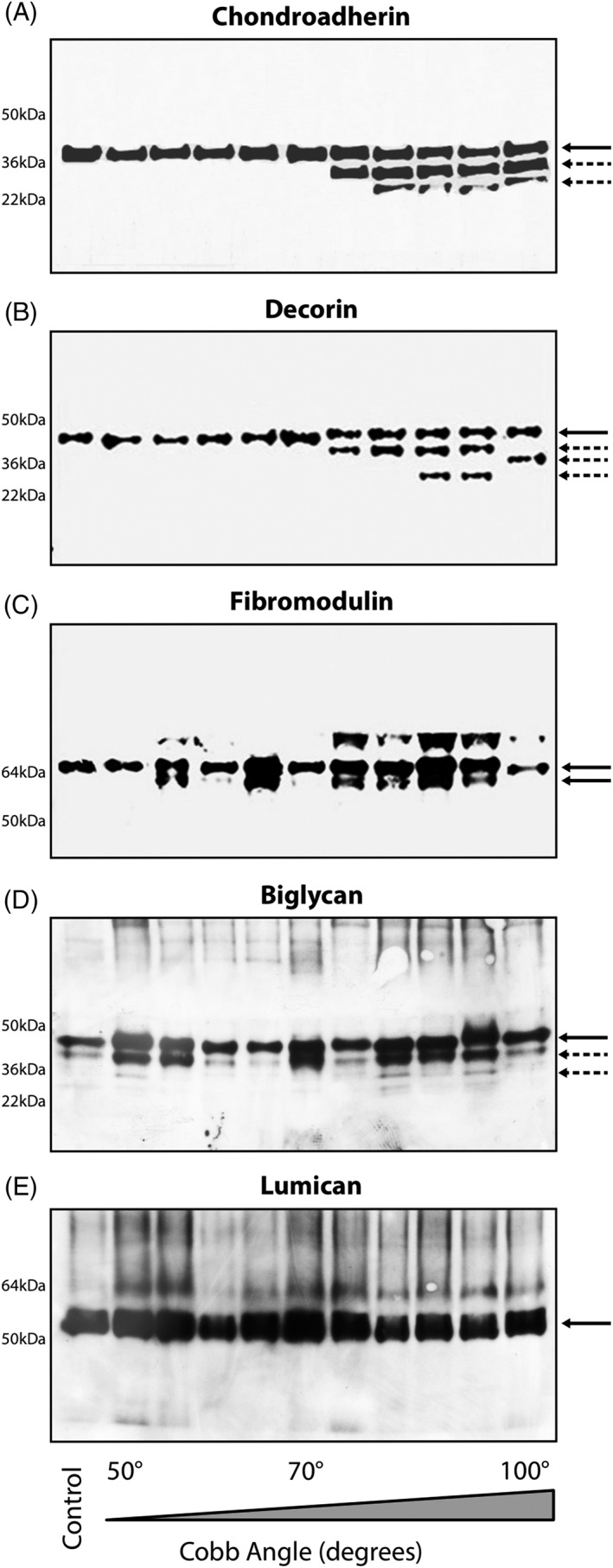
Western blot analysis of SLRPs on a 10% gel. Gels were loaded based on ascending grade of curvatures. The curves ranged from 50° to 100°; the first lane is occupied by non‐scoliotic, non‐degenerated age‐matched control sample. A, Chondroadherin core protein at 38 Kda & its fragments below the core protein are indicated by the arrow. B, Decorin core protein at 45 kDa with fragments. Note fragments for Decorin & Chondroadherin appear as the spinal curves magnitudes reach 70° of cobb angle. C, Doublet bands of Biglycan at 45 kDa along with its fragmented species. D, Double and single bands of fibromodulin appearing around 65 kDa. E, Non‐fragmented Lumican appears as single band at 55 kDa

Protein extracts of facet joint pairs (left and right facets) at the apex of the curve were pooled for 10 patients. The 10 scoliotic and 1 age‐matched non‐scoliotic patient samples were analyzed on a weight per volume basis by western blotting using antibodies for chondroadherin, decorin, biglycan and lumican (Figure 5).

### 3.5 Decorin and chondroadherin

Fragmentation of decorin and chondroadherin appeared as curve severity progressed (Figure 5). Chondroadherin core protein appeared at 38 kDa with 1 or 2 shorter fragments prominent between 22 and 35 kDa. Fragments appeared when the scoliotic curve progressed above 70° of Cobb angle (Figure 5A)**.** Similarly, decorin core protein appeared at 45 kDa, with 1 or 2 prominent fragments between 22 and 36 kDa, (Figure 5B)**.**

### 3.6 Biglycan, Lumican, and Fibromodulin

Biglycan fragmentation was present in all samples irrespective of curvature severity. For all samples, biglycan core protein appeared as a prominent band at around 45 kDa, with fragments evident at 36 kDa in all samples. A second 22 kDa fragment appeared mainly in curvatures above 70° (Figure 5C)**.** Fibromodulin core protein was apparent as a single or double band around 65 kDa (Figure 5D). Lumican appeared as a single band at 55 kDa with a faint lower molecular size fragment in a few samples (Figure 5E).

## 4 DISCUSSION

Articular facet joints in AIS patients demonstrated multiple signs of tissue deterioration which occurs in the form of proteoglycan loss, increased cellular proliferation, overexpression of MMP‐3, −13, and IL‐1ß. The tissue also presents a distinct fragmentation of SLRPs, especially chondroadherin and decorin, with curves characterized by a bigger Cobb angle. The results from this study thus suggest that young facet joint cartilage tissues in AIS patients undergo changes comparable to aged individuals with osteoarthritis.

As the articular cartilage matures after epiphyseal closure around 20 years of age, its proteoglycan content decreases, and the tissue slowly degrades with aging, a process that is strongly enhanced in OA.^24^ In young scoliotic patients who were less than 20 years of age, loss of proteoglycan from the superficial zone was evident, and distinctive fragmentation of SLRPs was apparent confirming an early onset of facet degradation in AIS. This is somewhat similar to IVD matrix disruption as we have previously reported for chondroadherin fragmentation in AIS IVDs^25^, ^26^ and the recently reported aggrecan fragmentation in AIS IVDs.^27^

In articular cartilage, the histological progression of OA is evidenced by surface discontinuity, fibrillation, and erosion (Figure 1E)**.** These characteristic changes were evaluated by a modified OARSI grading^20^ for the analysis of the AIS and non‐scoliotic facet articular cartilage. This grading system shows that scoliotic tissues had the strongest OA‐related phenotype with significantly higher OARSI grade compared to the control group. Furthermore, proteoglycan loss in facet joints of AIS patients was comparable to affected joints in osteoarthritic patients. In fact, the average age of the scoliotic donors was 15 and that of cadaveric organ donors was 34 years, which further reinforces the finding that known age‐related OA‐associated changes were more pronounced in younger AIS facets. Interestingly, the only age‐matched non‐scoliotic donor (17 years old) displayed similar amounts of proteoglycan content and OA‐related changes to the older non‐scoliotic donors, suggesting that the difference seen between control and scoliotic groups are not solely age‐related.

There are many previous studies describing results in the context of a simple 2‐dimensional concave or convex side of the spine. However, a simple 2‐dimensional analysis by x‐ray of spinal concavity and convexity cannot be used to assess differential biomechanical loads across facet joints for two reasons: (1) It does not account for spinal segment rotation which affects the perceived load on each side of the vertebrae and (2) Contrary to IVDs where the concave side gets wedged during scoliosis, the facet joints are not oriented perpendicularly to the spine, which alters the load‐bearing as well. For example, the Lenke 1 facet joints showed advanced deterioration on the concave side (Figure 2). However, for more complex curves like the Lenke 3, a different pattern is observed (Figure 2). These observations are in accordance with recent studies that found the higher loading on facet joints in degenerative lumbar scoliosis is dependent on the curve intensity, the position of the apex, and spinal movements, which do not always correlate with the concavity of the curvature.^28^ For these reasons, the scoliotic facet joints in this study were all considered as abnormally loaded, and they were compared to the cadaveric non‐scoliotic facet joints which we deemed being exposed to physiological and balanced loading. An in‐depth 3‐dimensional analysis is needed to decipher the link between biomechanical forces and tissue deterioration.

Although osteoarthritis is an age‐related disease,^29^ there exist other characteristic features that separate osteoarthritis from normal age‐related changes. These include cellular proliferation, overexpression of matrix‐degrading enzymes, and increased pro‐inflammatory factors.^30^ We found a significantly higher (*P* < .0001) cellularity in scoliotic cartilage, which was expected because of the age difference between groups and because cell density in articular cartilage decreases with age. However, since the cell density decrease is not linear during development and the bulk of the cell loss is seen before adolescence,^31^ we believe the high cell density in scoliotic cartilage is abnormal. To support this argument, the only truly age‐matched non‐scoliotic donor (17 years old) included in our study had lower cell density than 79% of the scoliotic samples. (Figure 3A) Interestingly, when single donor scoliotic facet joint pairs from one vertebra are separated and compared one side to the other, there is a constant difference in cell density. This difference is significant (*P* < .05) when all scoliotic facet joint pairs are separated into “high‐cellularity” and “low‐cellularity” subgroups. This separation showed a 1.6‐fold difference in cell numbers between the two subgroups. However, this difference is not seen in non‐scoliotic cartilage, which has very similar cellularity on both sides of a facet joint pair. We believe that this is due to the differential load applied the facet joint pairs when the spine is curved in scoliosis. This hypothesis also needs biomechanical analysis to be validated. To determine the cause of a high cell density and to potentially uncover an osteoarthritic marker in cell proliferation, we performed immunohistochemistry of the proliferative marker Ki‐67. Scoliotic cartilage had significantly (*P* < .05) more proliferating cells, suggesting that the higher cell count in these samples is in fact due to proliferation. Again, the one age‐matched non‐scoliotic donor (17 years old) had 20% proliferating cells compared to the scoliotic donors’ 80%, which can be used to exclude the age difference as an explanation. Clusters of cells resulting from proliferation are a histological hallmark of osteoarthritis and were of common occurrence (Figure 3C) in scoliotic facet joints. However, cell clusters were not always present,^30^, ^32^ suggesting the possibility that mechanisms other than proliferation, such as cell migration, could have been associated with increased cell density. The chondrons in abnormally loaded cartilage can become distended as the matrix is shifting under forces. This triggers mechanotransductive signals to the chondrocytes which are driven to proliferate and fill up the larger chondrons, which could be a potential cause seen in the scoliotic samples we studied (Figure 3C).^33^

Tissue homeostasis is critical in maintaining healthy cartilage. If the balance shifts toward matrix catabolism, the cartilage will quickly lose proteoglycans, water content, and consequentially its load‐bearing function. This is seen in osteoarthritis when hypertrophic chondrocytes start overproducing matrix‐degrading enzymes and pro‐inflammatory factors in response to an inflammatory event. Here, we focused on 4 predominant degenerative factors that are overexpressed in cartilage degradation: proteases MMP3, MMP13, and inflammatory cytokines IL‐1ß and IL‐6.^34^, ^35^ Immunohistochemistry revealed an upregulation of the two proteases of interest, MMP3 and MMP13, compared to the non‐scoliotic control group. The biggest difference was seen in MMP13, which had on average 2‐fold more positive cell staining. These results follow the previous findings in which MMP‐13 was found to be the most upregulated protease in OA.^36^ In accordance to the previous findings, the pro‐inflammatory cytokine IL‐1ß was upregulated which is known to induce MMP secretion in articular cartilage.^37^ IL‐6, however, remained unchanged between the two groups, but its role in OA is also less understood.

Matrix‐degrading enzymes MMP ‐1,2,3,5,7,10,13 and HTRA1 effectively cleave most SLRPs, fibronectin, collagen, and proteoglycans .^16^, ^26^, ^38^ Overloaded cartilage loses its water content as a consequence of proteoglycan loss and synthesizes complex catabolic and degradative molecules which lead to increased fragmentation of its matrix molecules.^39^ In this study, we report an extensive proteolysis of the SLRPs chondroadherin and decorin, which specifically appeared only when the scoliotic curves progressed toward higher Cobb angles with greater complexities. The appearances of fragmented chondroadherin and decorin were not detected in tissue sample from an age‐matched control individual. Extensive fragments of biglycan appeared in all the tissues examined and did not associate with scoliotic curve severities. This suggests that biglycan may be more sensitive to tissue loading in comparison to the others SLRPs.^40^ The molecular mass of the chondroadherin, decorin, biglycan, and fibromodulin fragments were between 22 and 35 kDa, and based on previous reports, it can be suggested that proteolytic cleavage and abnormal loading in the scoliotic spine may have jointly contributed to the generation of these fragments.^26^, ^40^, ^41^ Lumican, on the contrary, was not fragmented in any sample.

As very little tissue‐specific details exist on the spinal curves in AIS patients, limitations are associated with this study: first, all the scoliotic facets examined in this study were subjected to distinct abnormal loading with variable patterns, which might explain the variable results in the scoliotic group. To assess this, biomechanical analysis would be required to better visualize and correlate the 3‐dimensional deformity to actual loading and cartilage degeneration. Second, the scarcity of young cadaveric donors influenced the age difference between the scoliotic and non‐scoliotic groups. However, this can be used to reinforce our conclusion by considering that cartilage degenerates with age, and the scoliotic tissues were already more degenerate than the older control group.

In conclusion, this study shows the degenerative effects of chronic abnormal loading on the articular facet joints of patients with AIS. In these tissues, we observed hallmarks of OA, such as proteoglycan loss, overexpression of pro‐inflammatory mediators, increased synthesis of matrix‐degrading proteases, and fragmentation of the SLRPs. As with patients with age‐related OA, the premature joint degeneration seen in scoliotic patients is likely to contribute to the pain perceived in some of these individuals.

## References

[1] Cheng JC , Castelein RM , Chu WC , et al. Adolescent idiopathic scoliosis. Nat Rev Dis Primers. 2015;1:15030. 27188385 10.1038/nrdp.2015.30

[2] Weinstein SL , Dolan LA , Cheng JCY , Danielsson A , Morcuende JA . Adolescent idiopathic scoliosis. Lancet. 2008;371:1527‐1537. 18456103 10.1016/S0140-6736(08)60658-3

[3] Floman Y , Burnei G , Gavriliu S , et al. Surgical management of moderate adolescent idiopathic scoliosis with ApiFix®: a short peri‐ apical fixation followed by post‐operative curve reduction with exercises. Scoliosis. 2015;10:4. 25685175 10.1186/s13013-015-0028-9 PMC4328564

[4] Danielsson AJ , Hasserius R , Ohlin A , Nachemson AL . A prospective study of brace treatment versus observation alone in adolescent idiopathic scoliosis: a follow‐up mean of 16 years after maturity. Spine. 2007;32:2198‐2207. 17873811 10.1097/BRS.0b013e31814b851f

[5] Lenke LG , Edwards CC 2nd , Bridwell KH . The Lenke classification of adolescent idiopathic scoliosis: how it organizes curve patterns as a template to perform selective fusions of the spine. Spine (Phila Pa 1976). 2003;28:S199‐S207. 14560193 10.1097/01.BRS.0000092216.16155.33

[6] Kettler A , Wilke H‐J . Review of existing grading systems for cervical or lumbar disc and facet joint degeneration. Eur Spine J. 2006;15:705‐718. 16172902 10.1007/s00586-005-0954-y PMC3489462

[7] Adams MA , McNally DS , Dolan P . 'Stress' distributions inside intervertebral discs. The effects of age and degeneration. J Bone Joint Surg Br. 1996;78:965‐972. 8951017 10.1302/0301-620x78b6.1287

[8] Stokes IAF . Mechanical function of facet joints in the lumbar spine. Clin Biomech. 1988;3:101‐105. 10.1016/0268-0033(88)90052-6 23915841

[9] Sophia Fox AJ , Bedi A , Rodeo SA . The basic science of articular cartilage: structure, composition, and function. Sports Health. 2009;1:461‐468. 23015907 10.1177/1941738109350438 PMC3445147

[10] Felson DT , Lawrence RC , Dieppe PA , et al. Osteoarthritis: new insights. Part 1: the disease and its risk factors. Ann Intern Med. 2000;133:635‐646. 11033593 10.7326/0003-4819-133-8-200010170-00016

[11] Andriacchi TP , Mündermann A . The role of ambulatory mechanics in the initiation and progression of knee osteoarthritis. Curr Opin Rheumatol. 2006;18:514‐518. 16896293 10.1097/01.bor.0000240365.16842.4e

[12] Guilak F . Biomechanical factors in osteoarthritis. Best Pract Res Clin Rheumatol. 2011;25:815‐823. 22265263 10.1016/j.berh.2011.11.013 PMC3266544

[13] Kalichman L , Li L , Kim D , et al. Facet joint osteoarthritis and low back pain in the community‐based population. Spine. 2008;33:2560‐2565. 18923337 10.1097/BRS.0b013e318184ef95 PMC3021980

[14] Loeser RF . Molecular mechanisms of cartilage destruction: mechanics, inflammatory mediators, and aging collide. Arthritis Rheum. 2006;54:1357‐1360. 16645963 10.1002/art.21813 PMC1774815

[15] Merline R , Schaefer RM , Schaefer L . The matricellular functions of small leucine‐rich proteoglycans (SLRPs). J Cell Commun Signal. 2009;3:323‐335. 19809894 10.1007/s12079-009-0066-2 PMC2778586

[16] Melrose J , Fuller ES , Roughley PJ , et al. Fragmentation of decorin, biglycan, lumican and keratocan is elevated in degenerate human meniscus, knee and hip articular cartilages compared with age‐matched macroscopically normal and control tissues. Arthritis Res Ther. 2008;10:R79. 18620607 10.1186/ar2453 PMC2575625

[17] Heinegård D , Saxne T . The role of the cartilage matrix in osteoarthritis. 2010;7:50. 10.1038/nrrheum.2010.198 21119607

[18] Vogl T , Eisenblätter M , Völler T , et al. Alarmin S100A8/S100A9 as a biomarker for molecular imaging of local inflammatory activity. Nat Commun. 2014;5:4593. 25098555 10.1038/ncomms5593 PMC4143994

[19] Haglund L , Bernier SM , Önnerfjord P , Recklies AD . Proteomic analysis of the LPS‐induced stress response in rat chondrocytes reveals induction of innate immune response components in articular cartilage. Matrix Biol. 2008;27:107‐118. 18023983 10.1016/j.matbio.2007.09.009

[20] Hoemann C , Kandel R , Roberts S , et al. International cartilage repair society (ICRS) recommended guidelines for histological endpoints for cartilage repair studies in animal models and clinical trials. Cartilage. 2011;2:153‐172. 26069577 10.1177/1947603510397535 PMC4300784

[21] Pritzker KP , Gay S , Jimenez SA , et al. Osteoarthritis cartilage histopathology: grading and staging. Osteoarthritis Cartilage. 2006;14:13‐29. 16242352 10.1016/j.joca.2005.07.014

[22] Lee S . Method for Quantitative Analysis of Glycosaminoglycans and Type II Collagen in Chondrocyte‐seeded Articular Cartilage Scaffolds with Varied Cross‐linking Density. Cambridge, MA, Massachusetts Institute of Technology; 2005.

[23] Sandell LJ , Aigner T . Articular cartilage and changes in arthritis. An introduction: cell biology of osteoarthritis. Arthritis Res. 2001;3:107‐113. 11178118 10.1186/ar148 PMC128887

[24] Triphaus GF , Schmidt A , Buddecke E . Age‐related changes in the incorporation of [35S]sulfate into two proteoglycan populations from human cartilage. Hoppe‐Seyler's Zeitschrift Physiol Chemie. 1980;361:1773‐1779. 10.1515/bchm2.1980.361.2.1773 7461605

[25] Haglund L , Ouellet J , Roughley P . Variation in chondroadherin abundance and fragmentation in the human scoliotic disc. Spine (Phila Pa 1976). 2009;34:1513‐1518. 19525844 10.1097/BRS.0b013e3181a8d001

[26] Akhatib B , Önnerfjord P , Gawri R , et al. Chondroadherin fragmentation mediated by the protease HTRA1 distinguishes human intervertebral disc degeneration from normal aging. J Biol Chem. 2013;288:19280‐19287. 23673665 10.1074/jbc.M112.443010 PMC3696698

[27] Shu CC , Melrose J . The adolescent idiopathic scoliotic IVD displays advanced aggrecanolysis and a glycosaminoglycan composition similar to that of aged human and ovine IVDs. Eur Spine J. 2018; epub ahead of print. 10.1007/s00586-018-5515-2 29441417

[28] Wang L , Zhang B , Chen S , Lu X , Li ZY , Guo Q . A validated finite element analysis of facet joint stress in degenerative lumbar scoliosis. World Neurosurg. 2016;95:126‐133. 27521732 10.1016/j.wneu.2016.07.106

[29] Anderson AS , Loeser RF . Why is osteoarthritis an age‐related disease? Best Pract Res Clin Rheumatol. 2010;24:15‐26. 20129196 10.1016/j.berh.2009.08.006 PMC2818253

[30] Lotz MK , Otsuki S , Grogan SP , Sah R , Terkeltaub R , D'Lima D . Cartilage cell clusters. Arthritis Rheum. 2010;62:2206‐2218. 20506158 10.1002/art.27528 PMC2921934

[31] Stockwell RA . The cell density of human articular and costal cartilage. J Anat. 1967;101:753‐763. 6059823 PMC1270909

[32] Athanasiou D , Aguilà M , Bevilacqua D , Novoselov SS , Parfitt DA , Cheetham ME . The cell stress machinery and retinal degeneration. FEBS Lett. 2013;587:2008‐2017. 23684651 10.1016/j.febslet.2013.05.020 PMC4471140

[33] Responte DJ , Lee JK , Hu JC , Athanasiou KA . Biomechanics‐driven chondrogenesis: from embryo to adult. FASEB J. 2012;26:3614‐3624. 22673579 10.1096/fj.12-207241 PMC3425829

[34] Yamamoto K , Okano H , Miyagawa W , et al. MMP‐13 is constitutively produced in human chondrocytes and co‐endocytosed with ADAMTS‐5 and TIMP‐3 by the endocytic receptor LRP1. Matrix Biol. 2016;56:57‐73. 27084377 10.1016/j.matbio.2016.03.007 PMC5146981

[35] Mathy‐Hartert M , Hogge L , Sanchez C , Deby‐Dupont G , Crielaard JM , Henrotin Y . Interleukin‐1beta and interleukin‐6 disturb the antioxidant enzyme system in bovine chondrocytes: a possible explanation for oxidative stress generation. Osteoarthritis Cartilage. 2008;16:756‐763. 18291685 10.1016/j.joca.2007.10.009

[36] Kevorkian L , Young DA , Darrah C , et al. Expression profiling of metalloproteinases and their inhibitors in cartilage. Arthritis Rheum. 2004;50:131‐141. 14730609 10.1002/art.11433

[37] Kobayashi M , Squires GR , Mousa A , et al. Role of interleukin‐1 and tumor necrosis factor alpha in matrix degradation of human osteoarthritic cartilage. Arthritis Rheum. 2005;52:128‐135. 15641080 10.1002/art.20776

[38] Jabłońska‐Trypuć A , Matejczyk M , Rosochacki S . Matrix metalloproteinases (MMPs), the main extracellular matrix (ECM) enzymes in collagen degradation, as a target for anticancer drugs. J Enzyme Inhib Med Chem. 2016;31:177‐183. 27028474 10.3109/14756366.2016.1161620

[39] Sofat N . Analysing the role of endogenous matrix molecules in the development of osteoarthritis. Int J Exp Pathol. 2009;90:463‐479. 19765101 10.1111/j.1365-2613.2009.00676.x PMC2768145

[40] Brown S , Melrose J , Caterson B , Roughley P , Eisenstein SM , Roberts S . A comparative evaluation of the small leucine‐rich proteoglycans of pathological human intervertebral discs. Eur Spine J. 2012;21:154‐159. 10.1007/s00586-012-2179-1 PMC3326086 22358337

[41] Melrose J , Smith SM , Fuller ES , et al. Biglycan and fibromodulin fragmentation correlates with temporal and spatial annular remodelling in experimentally injured ovine intervertebral discs. Eur Spine J. 2007;16:2193‐2205. 17899219 10.1007/s00586-007-0497-5 PMC2140141

